# Identification of Cell Surface Proteins as Potential Immunotherapy Targets in 12 Pediatric Cancers

**DOI:** 10.3389/fonc.2012.00194

**Published:** 2012-12-17

**Authors:** Rimas J. Orentas, James J. Yang, Xinyu Wen, Jun S. Wei, Crystal L. Mackall, Javed Khan

**Affiliations:** ^1^Immunology Section, Pediatric Oncology Branch, Center for Cancer Research, National Cancer Institute, National Institutes of HealthBethesda, MD, USA; ^2^Oncogenomics Section, Advanced Technology Center, Pediatric Oncology Branch, Center for Cancer Research, National Cancer Institute, National Institutes of HealthGaithersburg, MD, USA

**Keywords:** cancer antigens, rhabdomyosarcoma, Ewing’s sarcoma, osteosarcoma, hepatoblastoma, glioblastoma, neuroblastoma, sarcoma

## Abstract

Technological advances now allow us to rapidly produce CARs and other antibody-derived therapeutics targeting cell surface receptors. To maximize the potential of these new technologies, relevant extracellular targets must be identified. The Pediatric Oncology Branch of the NCI curates a freely accessible database of gene expression data for both pediatric cancers and normal tissues, through which we have defined discrete sets of over-expressed transcripts in 12 pediatric cancer subtypes as compared to normal tissues. We coupled gene expression profiles to current annotation databases (i.e., Affymetrix, Gene Ontology, Entrez Gene), in order to categorize transcripts by their sub-cellular location. In this manner we generated a list of potential immune targets expressed on the cell surface, ranked by their difference from normal tissue. Global differences from normal between each of the pediatric tumor types studied varied, indicating that some malignancies expressed transcript sets that were more highly diverged from normal tissues than others. The validity of our approach is seen by our findings for pre-B cell ALL, where targets currently in clinical trials were top-ranked hits (CD19, CD22). For some cancers, reagents already in development could potentially be applied to a new disease class, as exemplified by CD30 expression on sarcomas. Moreover, several potential new targets shared among several pediatric solid tumors are herein identified, such as MCAM (MUC18), metadherin (MTDH), and glypican-2 (GPC2). These targets have been identified at the mRNA level and are yet to be validated at the protein level. The safety of targeting these antigens has yet to be demonstrated and therefore the identified transcripts should be considered preliminary candidates for new CAR and therapeutic antibody targets. Prospective candidate targets will be evaluated by proteomic analysis including Westerns and immunohistochemistry of normal and tumor tissues.

## Introduction

“Tumor-associated antigens” are multi-faceted and can be defined as any entity that the immune system can avail itself of to protect the host from disease. Virus-encoded tumor antigens are currently being targeted by preventive vaccines, such as for papilloma viruses, or by adoptive immunotherapy, as in EBV-associated post-transplant lymphoma. Self-antigens presented on major histocompatibility antigens (MHC) molecules are also key to clearing disease by donor-derived cells in the context of bone-marrow or hematopoietic stem cell transplantation (HSCT; Miller et al., 2010). However, with the advent of antibody-based therapies, cell-surface antigens on tumor cells can be targeted without first requiring processing and presentation by the MHC. Thus, a tumor antigen may be a unique molecule expressed by a tumor that is not encoded by the healthy genome, a non-mutated developmental antigen now re-expressed on a tumor cell, or a self-antigen than can be safely targeted without loss of host integrity. One example of recent interest is the re-expression of the developmentally regulated ALK protein on neuroblastoma (Mosse et al., 2008). The work we present here indicates that there are other such targets that remain to be discovered.

Our goal was to develop a method for identifying tumor antigen candidates that could be targeted by antibody or CAR-based therapies by leveraging publically available microarray gene expression databases of pediatric cancer. Previously we explored a series of established xenograft cell lines from pediatric cancers in support of the Pediatric Oncology Pre-clinical Protein-Tissue Array Project (POPP-TAP), a project jointly supported by the Children’s Oncology Group and the NCI (Whiteford et al., 2007). In analyzing these xenograft models we also began to assemble comparator normal tissue databases^1^ (“Pediatric Xenograft & Tumor Gene Expression Database”). This earlier study was carried out using a gene expression array from Research Genetics (Huntsville, AL, USA). These studies were expanded to include normal tissue analysis on the commonly available Affymetrix platform (“Pediatric Tumor Affymetrix Database” at see text footnote 1), allowing for easier comparison to analyses from other groups (Chen et al., 2008). Averaging gene expression levels from individual tumor samples according to diagnostic category, and comparing this expression level transcript by transcript to average normal tissue expression levels, allowed a statistical measure of the difference of that transcripts expression from its expression in normal tissue. Ranking the identified transcripts, and then filtering them for plasma membrane expression is a first step in the high-throughput identification of all available targets on the surface of pediatric cancers. We propose that this approach will be especially valuable in solid tumors, as a paucity of well-described targets remains a challenge to the field. The data we present here should allow for the rapid assessment of these target antigens for their suitability as candidates for immunotherapy.

## Materials and Methods

### Expression database for pediatric tumors

The Pediatric Tumor Affymetrix Database is freely accessible and can be found on-line at the NCI Pediatric Oncology Branch Oncogenomics Section web-site^2^. The gene chip used was U133 P2 (Affymetrix, Santa Clara, CA, USA).

### Construction of an algorithm to identify candidate membrane proteins following disease-specific analysis of gene expression

The design of this program was broken down into stages. First, annotation data from several public databases were collated and used to identify cell surface proteins. This included Gene Ontology^3^, Affymetrix chip data references, the Human Protein Reference Database^4^, and the primary literature. Second, a database was constructed using this data, along with our gene expression data, to store the data in a usable format for analysis in MySQL, using a set of Python scripts to automate core database functions. Using this database, an auxiliary table was built, combining gene expression levels over tissue sample categories, corresponding specifically to samples from indicated pediatric cancers, using a two-sampled *t*-test (implemented using the SciPy Python package for scientific and statistical functions) with all samples in each category tested against a series of normal tissue samples (expression profiles of lung, liver, kidney, heart, adrenal, cerebrum, cerebellum, uterus, testes, stomach, spleen, bladder, skeletal muscle, prostate, and ovary, previously described in; Whiteford et al., 2007) in order to generate a *T*-statistic and *p* value, scoring each gene’s expression level in each cancer type vs. normal expression levels. Genes in this auxiliary table were then sorted in order of descending differential expression.

## Results

### Tumors analyzed

We restricted our current analysis to the 12 pediatric tumor types that had more than five samples available in the Pediatric Tumor Affymetrix Database: Pre-B Acute Lymphocytic Leukemia (Pre_B_ALL), Embryonal Rhabdomyosarcoma (ERMS), Alveolar Rhabdomyosarcoma (ARMS), Soft-Tissue Sarcoma (STS) that is not classified as Rhabdomyosarcoma (Non-RMS_STS or simply STS), Desmoplastic Small Round Cell Tumor (DSRCT), Ewing’s Sarcoma (EWS), Alveolar Soft Part Sarcoma (ASPS), Glioblastoma (GBM), Osteosarcoma (OS), Neuroblastoma-MYCN-amplified (NBL_MA, MYCNA-NBL), Neuroblastoma non-MYCN-amplified (NBL), and Hepatoblastoma (HBL). Some well-known tumors, like Wilm’s tumor, could not yet be included; nevertheless, these 12 types represent the majority of all pediatric solid tumors, and also includes the most common hematologic malignancy of children.

### Candidate antigens

We present here Pre_B_ALL as an example to demonstrate how data mining searches were organized. A standard *t*-test was used to compare the average gene expression signal from tumor vs. the set of normal tissues analyzed in the database. The normal tissue data was used as an aggregate average expression score per each query. The algorithm was also set to report out a *p* value, while filtering for surface membrane expression to define the targets of interest. We initially calculated *t-*test values > 10, and ordered the output to select for the highest *T* values. This process was repeated in a similar manner for each disease category. Table 1 shows the number of hits for each disease type in the database returned when this arbitrary *T* threshold of >10 was selected. A wide range of hits was returned, with some diseases like ARMS having 62 hits score above 10, while DSRCT had 0. This does not mean DSRCT has no significant hits, as a *T*-statistic of 10 is a very high-value. Rather it illustrates that on a global level each malignancy has developed its own phenotypic “distance” from the normal cell surface landscape.

**Table 1:** **Tumor-associated membrane proteins identified with a *T* value greater than 10**.

**Disease**	**Sample size**, *n* =	**Hits**
Pre-B ALL	9	120
ARMS	12	62
ASPS	7	52
Glioblastoma	7	52
NBL-MYCN-amplified	24	44
Non-RMS-STS	6	38
Osteosarcoma	17	31
Hepatoblastoma	7	24
Ewing’s sarcoma	19	22
ERMS	9	16
Neuroblastoma (NBL)	15	4
DSRCT	8	0

*The table lists the 12 disease categories analyzed and the number of individual gene expression profiles for each disease type currently featured in the Oncogenomics database. “Hits” refers to the number of tumor-associated membrane antigens reported whose *T* value is > 10 in comparison to normal*.

A major challenge for our approach is that annotation of membrane-associated protein expression has not (or perhaps currently cannot) been validated for accuracy. On-line programs such as the highly sophisticated TMHMM package^5^ can predict transmembrane structure, but cannot assign sub-cellular localization. The most extensive and accurate protein database groups (for example the Human Protein Reference Database/Pandey Lab^6^) are hand-annotating proteins and tracing them to the original literature in order to define sub-cellular localization. Therefore, we also had to utilize this approach and individually examine each membrane protein hit yielded by our algorithm by searching the available primary literature, primarily using Gene hosted by NCBI^7^, to determine if the “membrane” tag associated with a transcript’s annotation truly denotes the extracellular plasma membrane. If an antigen is not expressed at the surface, that antigen will not be useful for immune targeting as we have described. We thus excluded proteins restricted to the mitochondria, nuclear membrane, Golgi, endoplasmic reticulum, sorting vesicles, and other intracellular membrane-bound bodies. Membrane proteins expressed both on the surface and another sub-cellular compartment were included. Table 2 lists the top 25 extracellular membrane proteins for each disease type after individual annotation/inspection.

**Table 2:** **Top 25 pediatric cancer transcripts arranged by category**.

**CD antigens/immunology markers**
CD19, B cell marker
CD22, B cell marker
CD25, IL2RA
CD28
CD30, TNFRSF8
CD43, sialophorin
CD49D, ITGA4 (VLA-4)
CD53 (a tetraspanin)
CD72, B cell marker
CD73, NT5E
CD79A, B cell marker
CD79B, B cell marker
CD85k, LILRB4, ILT3, leukocyte Ig-like R, subfamily B, member 4
CD107a
CD112, PVRL2 poliovirus R-related 2
CD115, CSF1R, colony-stimulating factor 1 receptor
CD146, MCAM, MUC18
CD155, PVR, polio-virus receptor
CD185, CXCR5
CD204, MSR1, macrophage scavenger receptor 1
CD271, NGFR
CD276, B7-H3
CD279, PDCD1 (PD1)
CD280, MRC2, mannose R, C-type 2
CD281, TLR1, toll-like receptor 1
CD301 (CLEC10A)
CD353, SLAMF8
CD362, SDC2, syndecan 2
CKLF, chemokine-like factor
CLEC2D, C-type lectin domain family 2, member D (NK cells)
FLT3LG
GP1BB glycoprotein Ib, β polypeptide, CD42c
HLA-G
ICAM5, telencephalin
IGHA1/IgA1
IL1RAP, IL-1R accessory protein
IL17RE, Interleukin-17 receptor E
IL27RA, on EWS
MILR1, mast cell Ig-like receptor 1
MR1, MHC class 1-related
PTCRA, pre-TCR α
PODXL2, endoglycan, podocalyxin-like 2
PTPRCAP, CD45-AP
ULBP2, UL16 binding protein 2, NKG2D ligand 2, on OS
XG, Xg blood group
**Cell adhesion, cell junction**
AJAP1 Adherens Junction Associated (SHREW-1, binds CD147)
ASGR1, R2 (asialoglycoprotein receptor)
MILR1, mast cell Ig-like 1, Allergin-1
CADM1/IGSF4A, cell adhesion molecule 1
CADM4/IGSF4C
CDH15, cadherin 15, type 1, myotubule
CDH23, cadherin-related 23
CDHR5, MUCDHL
CELSR3, cadherin, EGF LAG seven-pass G-type receptor 3
CSPG4, chondroitin sulfate proteoglycan 4 (HMW-MAA, melanoma-associated)
FAT4, FAT tumor suppressor homolog 4, protocadherin CDHF14
GJA3, gap junction protein α 3 (connexin family)
GJB2, gap junction protein β 2
GPC2, glypican-2
IGSF9, Ig superfamily, member 9
LRFN4, leucine-rich repeat (LRR), and fibronectin type III domain containing 4
LRRN6A/LINGO1, LRR, and Ig domain containing 1
LRRC15, LRR containing 15
LRRC8E, LRR containing 8E
LRIG1, LRR, and Ig-like domains 1
LGR4, LRR containing G-protein-coupled receptor 4 (R-spondin receptor)
LYPD1, LY6/PLAUR domain containing 1
MARVELD2, MARVEL domain containing 2
MEGF10, multiple EGF-like domains 10, scavenger/phagocytic R
MPZL1, myelin protein zero-like 1
MTDH, metadherin
PANX3, pannexin 3
PCDHB10, protocadherin β 10
PCDHB12, protocadherin β 12
PCDHB13, protocadherin β 13
PCDHB18, protocadherin β 18 pseudogene
PCDHGA3, protocadherin γ subfam. A, 3
PERP, TP53 apoptosis effector
SGCB, sarcoglycan, β
VEZT, vezatin, adherens junctions transmembrane protein
**Enzymatic function**
DAGLB, diacylgycerol lipase β
SYT11, synaptogamin XI
WFDC10A, WAP four-disulfide core domain 10A, peptidase inhibitor, extracellular
**Growth factor R/development R**
ACVR2A, Activin R2A
ACVR2B, Activin R2B
EPHB2, ephrin receptor B2 (RTK)
EPHB3, ephrin receptor B3
EPHB4, ephrin receptor B4
EFNB1, ephrin-B1
EPOR, erythropoietin receptor
FGFR2, fibroblast growth factor receptor 2
FGFR4
GALR2, galanin receptor 2 (GPCR)
GLG1, Golgi glycoprotein 1, Cfr-1, cysteine rich FGFR, ESL-1, E-selectin ligand-1
GLP1R, glucagon-like peptide 1 R
HBEGF, heparin-binding EGF-like growth factor (cleaved at membrane)
IGF2R insulin-like growth factor-2 receptor
MET, met proto-oncogene, HGFR, hepatocyte growth factor receptor
UNC5C, unc-5 homolog C, a netrin R
VASN, vasorin, TGF-β R on vascular smooth muscle cells
DLL3, delta-like 3, a notch ligand
FZD10/CD350, frizzled homolog 10 (GPCR, Wnt pathway)
KREMEN2, kringle containing transmembrane protein 2 (DKK1 receptor)
TMEM198, MGC99813, required for Wnt signal transduction
NRG1, neuregulin 1
TMEFF1, transmembrane protein w/EGF-like, and two follistatin-like domains 1
**Neurotransmitter receptor**
ADRA2C, adrenergic A2C
CHRNA1, cholinergic R, nicotinic, α 1 (muscle)
CHRNB4, cholinergic R, nicotinic, β 4
CHRNA3, cholinergic R, nicotinic, α 3
CHRNG, cholinergic R, nicotinic, γ
DRD4, dopamine R D4, G-protein linked
GABRB3, GABA receptor, β3
GRIN3A, glutamate R, ionotropic, *N*-methyl-d-aspartate 3A
GRIN2C glutamate R, ionotropic, *N*-methyl d-aspartate 2C
GRIK4 glutamate R, ionotropic, kainate 4
HTR7 5-hydroxytryptamine (serotonin) R 7
SLC6A2, solute carrier family 6 (neurotransmitter, noradrenalin), member 2
SLC6A11, SLC6 (neurotransmitter transporter, GABA), member 11
SLC6A15, SLC (neutral amino acid transporter), member 15
SLC29A4, SLC29 (nucleoside transporters), member 4 (reuptake of monoamines into presynaptic neurons)
Ion channels, ion channel regulation
APT8B2 (ATPase, cation transport, phospholipid translocation)
NKAIN4 (C20orf58), Na+/K+ transporting ATPase interacting 4.
CACNA1A,Ca^2+^ channel, voltage-dependent, P/Q type, α 1A subunit
CACNA1B, Ca^2+^ channel, voltage-dependent, L type, α 1B subunit
CACNA1I, Ca^2+^ channel, voltage-dependent, α 1I subunit
CACNG8, Ca^2+^ channel, voltage-dependent, γ subunit 8
CACNG4, Ca^2+^ channel, voltage-dependent, γ subunit 4
CLCN7, chloride channel 7
NKAIN1, Na^+^/K^+^ transporting ATPase interacting 1, FMA77C
NKAIN4, Na^+^/K^+^ transporting ATPase interacting 4, C20orf58
KCNEA4, K^+^ voltage-gated channel, Isk-related family, member 4
KCNG2, K^+^ voltage-gated channel, subfamily G, member 2
KCNN3, K^+^ intermediate/small conductance Ca^2+^-activated channel, subfamily N, member 3
KCNQ2, K^+^ voltage-gated channel, KQT-like subfamily, member 2
KCNU1, K^+^ channel, subfamily U, member 1
P2RX3, purigenic receptor P2X, ligand-gated ion channel, 3
PKD1L2, polycystic kidney disease 1-like 2
PKD2L1, polycystic kidney disease 2-like 1
SLC9A1, SLC9 (Na^+^/H^+^ exchanger), member 1
SLC30A5, SLC30 (zinc transporter), member 5
SLC39A7, SLC39 (zinc transporter), member 7
SLC39A8, SLC39 (zinc transporter), member 8
TRPM4, transient receptor, potential cation channel, subfamily M, member 4
TRPV4, transient receptor, potential cation channel, subfamily V, member 4
TMEM16J/ANO9, anoctamin 9, Ca^2+^-activated Cl- channel
TMEM142B, ORAI2, ORAI Ca^2+^ release-activated Ca^2+^modulator 2
**SLC, solute carrier family members**
SLC6A15, SLC6 (neutral amino acid transporter), member 15
SLC5A8, SLC5 (iodide transporter), member 8
SLC7A1, SLC7 (cationic amino acid transporter, y+ system), member 1
SLC7A6, SLC7 (amino acid transporter light chain, y + L system), member 6
SLC10A3, SLC10 (sodium/bile cotransporter family), member 3
SLC10A4, SLC10 (sodium/bile acid cotransporter family), member 4
SLC13A5, SLC13 (sodium-dependent citrate transporter), member 5
SLC16A8, SLC16, member 8 (monocarboxylic acid transporter 3)
SLC19A1, FOLT, SLC19 (folate transporter), member 1
SLC35E2, SLC35, member E2
SLC38A6, SLC38, member 6
SLC38A9, SLC38, member 9 (putative sodium-coupled neutral amino acid transporter 9)
SLC43A3, EEG1 (embryonic epithelial gene-1), SLC43, member 3
**G-protein, G-protein coupled, or associated receptor**
ADORA2B, Adenosine R
BAI1 (brain angiogenesis inhibitor 1)
EDG6, S1PR4, sphingosine-1-phosphate R4
GPR1, G-coupled protein receptor 1
GPR26
GPR34
GPR44, PTGDR2 prostaglandin D2 receptor 2
GPR56
GPR68 (OGR-1, ovarian cancer G-protein-coupled receptor 1)
GPR175, TPRA1 transmembrane protein, adipocyte associated 1
LGR4, LRR containing G-protein-coupled receptor 4
MMD, monocyte macrophage differentiation-associated (has 7 transmembrane domains, not proven as a GPCR)
NTSR2, neurotensin receptor 2
OPN3, opsin 3
OR2L2, olfactory R, family 2, subfamily L, member 2
OSTM1, osteopetrosis associated transmembrane protein 1
P2RY8, purinogenic R P2Y, G-coupled, 8
P2RY11, purinogenic R P2Y, G-coupled, 11
PTGE3, prostaglandin E R 3 (subtype EP3)
SSTR5, somatostatin R 5
TBXA2R, thromboxane A2 receptor
**Migration/metastasis/motility**
ADAM22
CST11, cystatin 11 (epididymal specific)
MMP14, matrix metallopeptidase 14
LPPR1, RP11-35N6.1, lipid phosphate phosphatase-related protein type 1
LPPR3, PRG2 (plasticity related gene 2), LPPR type 3
LPPR5, PAP2D, PRG5, LPPR type 5
SEMA6B, semaphorin 6B
**Tetraspanin**
ALS2CR4 (amyotrophic lateral sclerosis 2 [juvenile])
LEPROTL1, leptin R overlapping transcript-like 1
MS4A4A, membrane-spanning, 4-domains, subfamily A, member 4A, CD20L1
MS4A6A, membrane-spanning 4-domains, subfamily A, member 6A, CD20L3
ROM1, retinal outer segment membrane protein 1
TM4SF5, transmembrane 4 L six family member 5
VANGL1, vang-like 1 (van gogh, Drosophila)
VANGL2, vang-like 2
**Uncharacterized**
C18orf1 (contains a LDLRA domain)
GSGL1, germ cell specific gene 1-like
ITM2A, integral membrane protein 2A
KIAA1715, LNP
LDLRAD3 (provisional), LDL receptor class A domain containing 3
ODZ3,odz Oz/ten-m homolog 3
SLC7A5P1 (LAT1-3TM) SLC7 (aminoacid transporter light chain, L system), member 5, psuedogene-1
STEAP1, six transmembrane epithelial antigen of the prostate 1

*The pediatric top 25. The membrane-bound proteins with the highest *T* value with respect to normal tissue expression are listed by disease type. ALL, Pre-B, Acute Lymphocytic Leukemia; ASPS, Alveolar Soft Part Sarcoma; DSRCT, Desmoplastic Small Round Cell Tumor; ERMS, Embryonal Rhabdomyosarcoma; ARMS, Alveolar Rhabdomyosarcoma; Non-RMS_STS or simply STS, Soft-Tissue Sarcoma that is not classified as Rhabdomyosarcoma; EWS, Ewing’s Sarcoma; GBM, Glioblastoma; OS, Osteosarcoma; NBL_MA, MYCNA-NBL, Neuroblastoma-MYCN-amplified; NBL, Neuroblastoma non-MYCN-amplified; HBL, Hepatoblastoma. This list was individually annotated to include only those transcripts whose proteins could be targeted from their extracellular aspect*.

**Figure 1:**
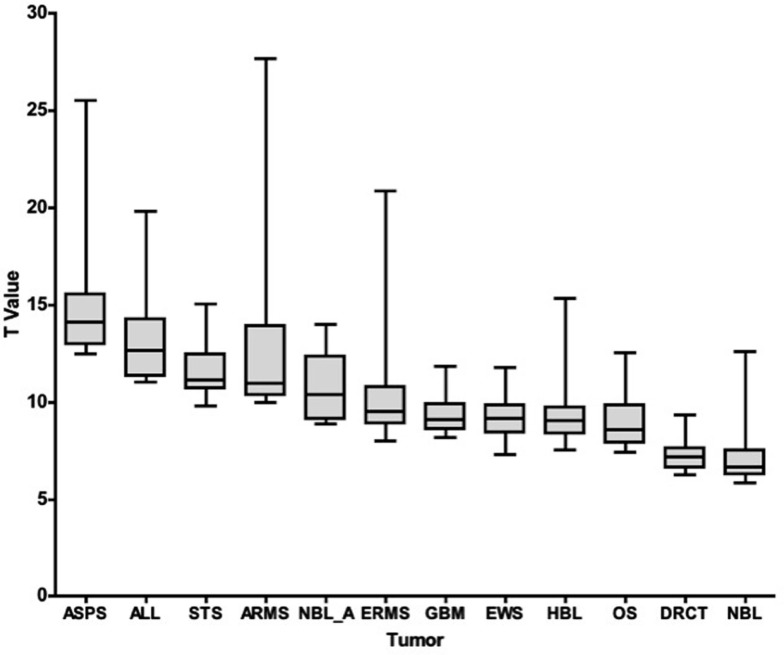
***T*-Statistic overview of the top 25 tumor-specific transcripts**. For each disease category (listed on the *x*-axis), the range of *T*-statistic values for the 25 membrane proteins most different from normal tissue, along with their average and quartiles, is represented as a box and whisker plot.

To compare global differences in the immune landscape for the 25 antigens most different from normal for each tumor type, we plotted the *T* value range of those 25 hits for each tumor type, Figure 1. When comparing the expression of a particular transcript in a tumor type versus normal tissue, we used a *T*-statistic and report the associated *p* value for that particular transcript (both with respect to difference from normal tissue). In looking at the top 25 hits for each tumor type, the lowest set of *T* values (that is membrane proteins that were least distinct from normal), were DSRCT and NBL. *T* values ranged from 9.3 to 6.9 for DSRCT and from 12.6 to 5.8 for NBL. The highest *T* values (tissues scoring the most different from normal) were seen for ASPS, Pre-B ALL, STS, and ARMS, which scored from 25.5 to 12.5, 19.8 to 11.0, 15.0 to 9.8, and 27.7 to 10.0, respectively. When *p* values were evaluated an essentially inverse pattern was seen; that is, high scoring *T* values had smaller (more significant) *p* values (not shown). These values demonstrate very good separation from normal and represent a set of targets that are important to further evaluate in each of these tumor types. As to the true immunogenicity of these tumor types, further studies are required to determine whether these differences can be accounted for by different strategies of immune escape or immune editing (Schreiber et al., 2011).

To better understand the antigens we have identified, we ordered the transcripts according to functional groups (Table 3). The first group consists of known CD antigens or immune marker proteins. The possession of a CD designation ensures that an antibody has been created to that transcript. The only known CD antigen we did not list in this section was CD222 (glucagon-like peptide 1 R), which was placed in the growth factor receptor category. In the CD antigen group, 12 out of 47 (appx. 25%) are expressed in pre-B ALL. This is representative of the tissue of origin, as CD antigens are often of immune cell origin. Nevertheless, many CD antigens are also expressed on other pediatric tumors.

**Table 3:** **Pediatric tumor antigens arranged by functional category**.

	Affy ID	Gene symbol	*T* value	*P* value	Gene name
**ALL (PRE-B)**					
1	206398_s_at	CD19	19.85	1.56E−15	CD19 molecule
2	205049_s_at	CD79A	17.90	1.33E−14	CD79a, immunoglobulin-associated alpha
3	205885_s_at	CD49D	16.64	6.01E−14	Integrin, alpha 4 (VLA-4)
4	205297_s_at	CD79B	16.18	1.06E−13	CD79b, immunoglobulin-associated beta
5	1568964_x_at	CD43	15.80	1.71E−13	Sialophorin (leukosialin)
6	222935_x_at	SLC39A8	14.78	6.59E−13	Solute carrier family 39 (zinc transporter)
7	209776_s_at	SLC19A1	13.84	2.47E−12	Solute carrier family 19 (folate transporter)
8	204960_at	CD45-AP	13.59	3.50E−12	PTPRCAP, CD45-associated protein
9	229686_at	P2RY8	13.57	3.61E−12	Purinergic R P2Y, G-protein coupled, 8 (fused w/CRLF2 in pre-B ALL (Mullighan’09)
10	214546_s_a	P2RY11	13.26	5.73E−12	Purinergic R P2Y, G-protein coupled, 11
11	236186_x_at	IL17RE	13.22	6.03E−12	Interleukin 17 receptor E
12	210176_at	CD281	12.70	1.32E−11	Toll-like receptor 1
13	206437_at	SIPR4	12.64	1.46E−11	Sphingosine-1-phosphate R 4, EDG6
14	215925_s_at	CD72	12.58	1.60E−11	CD72 molecule
15	212250_at	MTDH	12.47	1.89E−11	Metadherin
16	211042_x_at	CD146/MCAM	12.38	2.18E−11	Melanoma cell adhesion molecule (MUC18)
17	220132_s_at	CLEC2D	12.03	3.77E−11	C-type lectin domain family 2, member D
18	217513_at	MILR1	11.66	6.94E−11	Mast cell immunoglobulin-like receptor 1
19	208590_x_at	GJA3	11.38	1.09E−10	Gap junction protein, alpha 3, 46kDa
20	217422_s_at	CD22	11.33	1.18E−10	CD22 molecule
21	209574_s_at	C18orf1	11.29	1.26E−10	Chromosome 18 open reading frame 1
22	203578_s_at	SLC7A6	11.09	1.78E−10	SLC7 (cationic a. a. transporter), member 6
23	239422_at	GPC2	11.09	1.79E−10	Glypican 2 (cerebroglycan)
24	208299_at	CACNA1I	11.07	1.85E−10	Ca channel, voltage-dep., alpha 1I subunit
25	203416_at	CD53	11.05	1.91E−10	CD53 molecule
**ASPS**					
1	209963_s_at	EPOR	25.54	1.90E−20	Erythropoietin receptor
2	235197_s_at	OSTM1	24.96	3.45E−20	Osteopetrosis assoc. transmembr. protein 1
3	227393_at	ANO9	22.55	4.75E−19	Anoctamin 9 (TMEM16J)
4	244444_at	PKD1L2	18.60	6.34E−17	Polycystic kidney disease 1-like 2
5	202827_s_at	MMP14	18.42	8.12E−17	Matrix metallopeptidase 14
6	222379_at	KCNE4	15.65	4.62E−15	K voltage-gated channel, member 4
7	234985_at	LDLRAD3	15.45	6.23E−15	LDL receptor class A domain containing 3
8	206899_at	NTSR2	15.25	8.68E−15	Neurotensin receptor 2
9	219607_s_at	MS4A4A/CD20L1	15.07	1.16E−14	Membrane-spanning 4-domains, A4
10	230550_at	MS4A6A/CD20L3	14.78	1.83E−14	Membrane-spanning 4-domains, A6
11	38069_at	CLCN7	14.49	2.96E−14	Chloride channel 7
12	214830_at	SLC38A6	14.21	4.77E−14	Solute carrier family 38, member 6
13	206582_s_at	GPR56	14.14	5.32E−14	G protein-coupled receptor 56
14	201393_s_at	IGF2R/CD222	13.93	7.66E−14	Insulin-like growth factor 2 receptor
15	206545_at	CD28	13.49	1.63E−13	CD28 molecule
16	208552_at	GRIK4	13.48	1.65E−13	Glutamate receptor, ionotropic, kainate 4
17	219764_at	FZD10/CD350	13.34	2.12E−13	FZD10/frizzled homolog 10
18	223620_at	GPR34	13.03	3.69E−13	G protein-coupled receptor 34
19	210514_x_at	HLA-G	13.00	3.87E−13	HLA-G histocompatibility antigen, class I, G
20	214770_at	MSR1/CD204	12.86	5.05E−13	Macrophage scavenger receptor 1
21	206980_s_at	FLT3LG	12.79	5.70E−13	Fms-related tyrosine kinase 3 ligand
22	203104_at	CSF1R/CD115	12.76	6.06E−13	Colony stimulating factor 1 receptor
23	207565_s_at	MR1	12.58	8.32E−13	MHC, class I-related
24	213728_at	CD107a	12.50	9.76E−13	LAMP1
25	210692_s_at	SLC43A3	12.48	1.01E−12	Solute carrier family 43, member 3 (EEG)
**DRCT**					
1	212662_at	CD155	9.34	6.28E−09	Poliovirus receptor
2	211879_x_at	PCDHGA3	9.13	9.24E−09	Protocadherin gamma subfamily A, 3
3	206083_at	BAI1	8.81	1.71E−08	Brain-specific angiogenesis inhibitor 1
4	219692_at	KREMEN2	7.80	1.24E−07	Kringle containing transmembrane protein 2
5	214546_s_at	P2RY11	7.71	1.49E−07	Purinergic R P2Y, G-protein coupled, 11
6	215169_at	SLC35E2	7.66	1.63E−07	Solute carrier family 35, member E2
7	227281_at	SLC29A4	7.62	1.78E−07	SLC29 (nucleoside transporters), member 4
8	210651_s_at	EPHB2	7.51	2.22E−07	EPH receptor B2
9	204600_at	EPHB3	7.43	2.64E−07	EPH receptor B3
10	208215_x_at	DRD4	7.39	2.85E−07	Dopamine receptor D4
11	207634_at	CD279/PD1	7.38	2.91E−07	Programmed cell death 1
12	220798_x_at	LPPR3/PRG2	7.32	3.33E−07	Lipid phosphate phosphatase-related pr. 3
13	239422_at	GPC2	7.18	4.49E−07	Glypican 2 (cerebroglycan)
14	230668_at	NKAIN4	7.17	4.56E−07	Na++/K++ transporting ATPase interacting 4
15	206906_at	ICAM5	6.97	6.92E−07	Intercellular adhesion molecule 5
16	203149_at	CD112	6.82	9.71E−07	PVRL2, poliovirus receptor-related 2
17	204736_s_at	CSPG4	6.77	1.07E−06	Chondroitin sulfate proteoglycan 4
18	222293_at	CADM4/IGSF4C	6.69	1.29E−06	IGSF4C, Ig superfamily member 4C
19	206460_at	AJAP1	6.64	1.43E−06	Adherens junction assoc. prot. 1 (SHREW-1)
20	232415_at	PCDHB13	6.60	1.54E−06	Protocadherin beta 13
21	206341_at	CD25	6.57	1.66E−06	Interleukin 2 receptor, alpha
22	236186_x_at	IL17RE	6.45	2.16E−06	Interleukin 17 receptor E
23	208590_x_at	GJA3	6.40	2.42E−06	Gap junction protein, alpha 3, 46kDa
24	214605_x_at	GPR1	6.31	2.96E−06	G protein-coupled receptor 1
25	216680_s_at	EPHB4	6.29	3.10E−06	EPH receptor B4
**EWS**					
1	1554062_at	XG	11.76	3.69E−13	Xg blood group
2	212250_at	MTDH	10.10	1.76E−11	Metadherin
3	218989_x_at	SLC30A5	10.08	1.86E−11	SLC30 (zinc transporter), member 5
4	223675_s_at	VEZT	10.05	2.01E−11	Vezatin
5	205542_at	STEAP1	9.96	2.49E−11	6 transmembr. epithelial ag of the prostate 1
6	219427_at	FAT4	9.94	2.62E−11	FAT tumor suppressor homolog 4
7	220798_x_at	LPPR3/PRG2	9.78	3.90E−11	Lipid phosphate phosphatase-related prot. 3
8	239422_at	GPC2	9.51	7.60E−11	Glypican 2 (cerebroglycan)
9	235241_at	SLC38A9	9.51	7.64E−11	Solute carrier family 38, member 9
10	227933_at	LRRN6A/LINGO1	9.41	9.91E−11	Leucine rich repeat neuronal 6A
11	211042_x_at	CD146/MCAM	9.27	1.38E−10	Melanoma cell adhesion molecule (MUC18)
12	224392_s_at	OPN3	9.15	1.89E−10	Opsin 3 (encephalopsin, panopsin)
13	208299_at	CACNA1I	9.15	1.90E−10	Voltage-dependent, alpha 1I subunit
14	219360_s_at	TRPM4	9.11	2.11E−10	Transient R potential cation channel, M4
15	208550_x_at	KCNG2	8.71	5.86E−10	Voltage-gated channel, subfamily G2
16	212909_at	LYPD1	8.50	1.03E−09	LY6/PLAUR domain containing 1
17	205227_at	IL1RAP	8.50	1.04E−09	Interleukin 1 receptor accessory protein
18	214730_s_at	GLG1	8.44	1.22E−09	Golgi apparatus protein 1 (SEMA6C)
19	202747_s_at	ITM2A	8.41	1.30E−09	Integral membrane protein 2A
20	209768_s_at	CD42c	8.41	1.32E−09	GP1BB, glycoprotein Ib (platelet), beta
21	222062_at	IL27RA	8.34	1.56E−09	Interleukin 27 receptor, alpha
22	225717_at	KIAA1715	8.29	1.78E−09	KIAA1715 (Lnp, Lunapark)
23	202711_at	EFNB1	8.28	1.86E−09	Ephrin-B1
24	214830_at	SLC38A6	8.25	2.01E−09	Solute carrier family 38, member 6
25	236186_x_at	IL17RE	7.31	2.58E−08	Interleukin 17 receptor E
**GBM**					
1	208590_x_at	GJA3	11.85	1.69E−10	Gap junction protein, alpha 3, 46kDa
2	222935_x_at	SLC39A8	11.16	4.88E−10	SLC39 (zinc transporter), member 8
3	217573_at	GRIN2C	11.02	6.04E−10	Glutamate R, ionotropic, NMDA 2C
4	212250_at	MTDH	10.77	8.95E−10	Metadherin
5	236186_x_at	IL17RE	10.71	9.80E−10	Interleukin 17 receptor E
6	211226_at	GALR2	10.19	2.31E−09	Galanin receptor 2
7	205806_at	ROM1	9.72	5.12E−09	Retinal outer segment membrane protein 1
8	205891_at	ADORA2B	9.71	5.16E−09	Adenosine A2b receptor
9	232432_s_at	SLC30A5	9.64	5.83E−09	SLC30 (zinc transporter), member 5
10	208299_at	CACNA1I	9.50	7.47E−09	Ca channel, voltage-dependent, alpha 1I
11	222596_s_at	LGR4	9.47	7.87E−09	Leucine-rich repeat-containing GPCR4
12	208550_x_at	KCNG2	9.09	1.55E−08	K voltage-gated channel, subfamily G2
13	1553995_a_at	CD73	9.08	1.56E−08	5′-Nucleotidase, ecto
14	207048_at	SLC6A11	8.99	1.84E−08	SLC6 (GABA transporter), member 11
15	205120_s_at	SGCB	8.79	2.66E−08	Sarcoglycan, beta
16	235197_s_at	OSTM1	8.77	2.74E−08	Osteopetrosis assoc. transmembrane prot. 1
17	1556990_at	PERP	8.70	3.14E−08	PERP, TP53 apoptosis effector
18	223451_s_at	CKLF	8.68	3.25E−08	Chemokine-like factor
19	211042_x_at	CD146/MCAM	8.65	3.40E−08	Melanoma cell adhesion molecule (MUC18)
20	240140_s_at	LRIG1	8.61	3.69E−08	Leucine-rich repeats and Ig-like domains 1
21	214546_s_at	P2RY11	8.55	4.11E−08	Purinergic R P2Y, G-protein coupled, 11
22	208229_at	FGFR2	8.28	6.85E−08	Fibroblast growth factor receptor 2
23	202667_s_at	SLC39A7	8.26	7.09E−08	SLC39 (zinc transporter), member 7
24	209776_s_at	SLC19A1	8.26	7.13E−08	SLC19 (folate transporter), member 1
25	216464_x_at	PTGDR2	8.15	8.72E−08	Prostaglandin D2 receptor 2/CD294
**HBL**					
1	206242_at	TM4SF5	15.37	1.54E−12	Transmembrane 4 L six family member 5
2	212662_at	CD155	14.66	3.68E−12	Poliovirus receptor
3	235955_at	MARVELD2	12.90	3.75E−11	MARVEL domain containing 2
4	211576_s_at	SLC19A1	12.26	9.25E−11	SLC19 (folate transporter), member 1
5	214546_s_at	P2RY11	11.89	1.59E−10	Purinergic R P2Y, G-protein coupled, 11
6	206682_at	CD301	9.91	3.65E−09	C-type lectin domain family 10, member A
7	207634_at	CD279/PD1	9.61	6.20E−09	Programmed cell death 1
8	237273_at	KCNU1	9.55	6.82E−09	Potassium channel, subfamily U, member 1
9	206743_s_at	ASGR1	9.53	7.04E−09	Asialoglycoprotein receptor 1
10	223278_at	GJB2	9.27	1.11E−08	Gap junction protein, beta 2
11	220028_at	ACVR2B	9.23	1.20E−08	Activin A receptor, type IIB
12	209589_s_at	EPHB2	9.10	1.50E−08	EPH receptor B2
13	219796_s_at	CDHR5	9.06	1.62E−08	Cadherin-related family member 5
14	206130_s_at	ASGR2	8.83	2.45E−08	Asialoglycoprotein receptor 2
15	224392_s_at	OPN3	8.74	2.92E−08	Opsin 3 (encephalopsin, panopsin)
16	219386_s_at	CD353	8.53	4.23E−08	SLAM family member 8 (CD353)
17	227281_at	SLC29A4	8.44	5.04E−08	SLC29 (nucleoside transporters), member 4
18	1553995_a_at	CD73	8.41	5.35E−08	5′-Nucleotidase, ecto
19	211249_at	GPR68	8.40	5.42E−08	G protein-coupled receptor 68
20	228844_at	SLC13A5	8.33	6.18E−08	SLC13 (Na-dep. citrate transporter), 5
21	213728_at	CD107a	8.12	9.28E−08	LAMP1
22	211042_x_at	CD146/MCAM	7.99	1.19E−07	Melanoma cell adhesion molecule (MUC18)
23	207565_s_at	MR1	7.86	1.53E−07	MHC, class I-related
24	206361_at	PTGDR2	7.82	1.65E−07	Prostaglandin D2 receptor 2/CD294
25	219732_at	LPPR1/PRG3	7.56	2.79E−07	Lipid phosphate phosphatase-related prot. 1
**NB**					
1	239913_at	SLC10A4	12.60	4.66E−13	SLC10 (Na/bile acid cotransporter family), 4
2	210221_at	CHRNA3	9.39	3.75E−10	Cholinergic receptor, nicotinic, alpha 3
3	227281_at	SLC29A4	9.02	8.84E−10	SLC29 (nucleoside transporters), member 4
4	205122_at	TMEFF1	8.21	6.22E−09	TMEFF1
5	207516_at	CHRNB4	8.14	7.39E−09	Cholinergic receptor, nicotinic, beta 4
6	203414_at	MMD	7.75	1.93E−08	Monocyte to macrophage different.-assoc.
7	212290_at	SLC7A1	7.32	5.77E−08	SLC7 (cationic amino acid transporter), 1
8	218811_at	ORAI2	7.14	9.11E−08	ORAI Ca-release-activated Ca-modulator 2
9	221585_at	CACNG4	7.07	1.09E−07	Ca-channel, voltage-dep., gamma subunit 4
10	239422_at	GPC2	7.01	1.26E−07	Glypican 2 (cerebroglycan)
11	209198_s_at	SYT11	6.95	1.46E−07	Synaptotagmin XI
12	206343_s_at	NRG1	6.92	1.62E−07	Neuregulin 1
13	226029_at	VANGL2	6.65	3.26E−07	Vang-like 2 (van gogh, Drosophila)
14	219491_at	LRFN4	6.63	3.42E−07	Leucine rich repeat and fn type III domain,4
15	210353_s_at	SLC6A2	6.57	3.97E−07	SLC6 (noradrenalin transporter), member 2
16	219523_s_at	ODZ3	6.49	4.96E−07	Odz, odd Oz/ten-m homolog 3 (Drosophila)
17	1553956_at	TMEM237	6.42	5.96E−07	ALS2CR4, amyotrophic lateral sclerosis 2CR4
18	1552914_a_at	CD276	6.39	6.46E−07	CD276 molecule
19	220798_x_at	LPPR3/PRG2	6.31	7.97E−07	Lipid phosphate phosphatase-related pr. 3
20	219152_at	PODXL2	6.18	1.14E−06	Podocalyxin-like 2
21	40020_at	CELSR3	6.12	1.34E−06	Cadherin, EGF LAG seven-pass G-type R 3
22	206189_at	UNC5C	6.12	1.34E−06	Unc-5 homolog C (netrin receptor)
23	223854_at	PCDHB10	6.04	1.64E−06	Protocadherin beta 10
24	208118_x_a	SLC7A5P2	5.98	1.95E−06	SLC 7, member 5 pseudogene 2/IMAA
25	225832_s_at	DAGLBETA	5.86	2.65E−06	Diacylglycerol lipase beta
**NBL-MYCNA**					
1	239913_at	SLC10A4	14.03	2.18E−16	SLC10 (Na/bile acid cotransporter family), 4
2	205122_at	TMEFF1	13.74	4.15E−16	TMEFF1
3	219152_at	PODXL2	13.46	7.85E−16	Podocalyxin-like 2
4	210221_at	CHRNA3	13.12	1.74E−15	Cholinergic receptor, nicotinic, alpha 3
5	239422_at	GPC2	12.66	5.07E−15	Glypican 2 (cerebroglycan)
6	220798_x_at	LPPR3/PRG2	12.58	6.16E−15	Lipid phosphate phosphatase-related pr. 3
7	211042_x_at	CD146/MCAM	12.11	1.93E−14	Melanoma cell adhesion molecule (MUC18)
8	209776_s_at	SLC19A1	11.23	1.77E−13	SLC19 (folate transporter), member 1
9	210508_s_at	KCNQ2	11.11	2.43E−13	K voltage-gated channel, KQT-like, 2
10	203414_at	MMD	10.91	4.09E−13	Monocyte to macrophage different.-assoc.
11	219537_x_at	DLL3	10.43	1.45E−12	Delta-like 3
12	219438_at	NKAIN1	10.41	1.53E−12	Na + +/K + + transporting ATPase interacting 1
13	227281_at	SLC29A4	10.40	1.55E−12	SLC29 (nucleoside transporters), member 4
14	227690_at	GABRB3	10.35	1.80E−12	GABA, A receptor, beta 3
15	226029_at	VANGL2	10.09	3.62E−12	Vang-like 2 (van gogh, Drosophila)
16	242782_x_at	TMEM198	9.89	6.20E−12	Transmembrane protein 198
17	229276_at	IGSF9	9.39	2.46E−11	Immunoglobulin superfamily, member 9
18	1553956_at	TMEM237	9.25	3.69E−11	ALS2CR4, amyotrophic lateral sclerosis 2CR4
19	202594_at	LEPROTL1	9.13	5.15E−11	Leptin receptor overlapping transcript-like 1
20	212290_at	SLC7A1	9.07	6.08E−11	SLC7 (cationic a.a. transporter),1/LAT1-3TM
21	231397_at	LPPR5/PRG5	9.03	6.90E−11	PAP2D (phosphatidic acid phosphatase, 2)
22	232263_at	SLC6A15	8.98	7.98E−11	SLC6, member 15
23	1552914_a_at	CD276	8.94	8.90E−11	CD276 molecule (B7-H3)
24	219491_at	LRFN4	8.91	9.76E−11	LRFN4
25	207162_s_at	CACNA1B	8.86	1.12E−10	Ca channel, voltage-dep., L type, alpha 1B
**OS**					
1	202667_s_at	SLC39A7	12.57	1.75E−13	SLC39 (zinc transporter), member 7
2	240955_at	PANX3	11.06	4.14E−12	Pannexin 3
3	210087_s_at	MPZL1	10.96	5.22E−12	Myelin protein zero-like 1
4	212250_at	MTDH	10.92	5.69E−12	Metadherin
5	238542_at	ULBP2	10.11	3.58E−11	UL16 binding protein 2 (NKG2D)
6	218989_x_at	SLC30A5	9.93	5.34E−11	SLC30 (zinc transporter), member 5
7	206328_at	CDH15	9.78	7.67E−11	Cadherin 15, M-cadherin (myotubule)
8	209280_at	CD280	9.45	1.70E−10	MRC2, mannose receptor, C type 2
9	219330_at	VANGL1	9.18	3.21E−10	Vang-like 1 (van gogh, Drosophila)
10	202828_s_at	MMP14	9.07	4.21E−10	Matrix metallopeptidase 14
11	239433_at	LRRC8E	8.82	7.90E−01	Leucine rich repeat containing, 8E
12	218855_at	GPR175	8.67	1.13E−09	TPRA1
13	211042_x_at	CD146/MCAM	8.57	1.47E−09	Melanoma cell adhesion molecule (MUC18)
14	225867_at	VASN	8.44	2.04E−09	Vasorin
15	204928_s_at	SLC10A3	8.34	2.60E−09	SLC10 (Na/bile acid cotransporter fam.), 3
16	206399_x_at	CACNA1A	8.20	3.71E−09	Ca channel, voltage-dep. P/Q type, alpha 1A
17	203578_s_at	SLC7A6	8.00	6.31E−09	SPC7 (cationic amino acid transporter), 6
18	212662_at	CD155	7.98	6.53E−09	Poliovirus receptor
19	208299_at	CACNA1I	7.88	8.61E−09	Ca channel, voltage-dep., alpha 1I subunit
20	209030_s_at	CADM1/IGSF4A	7.88	8.66E−09	Immunoglobulin superfamily, member 4
21	213909_at	LRRC15	7.72	1.29E−08	Leucine rich repeat containing 15
22	205120_s_at	SGCB	7.54	2.09E−08	Sarcoglycan, beta
23	220455_at	SLC16A8	7.48	2.43E−08	SLC16, member 8
24	211837_s_at	PTCRA	7.43	2.77E−08	Pre T-cell antigen receptor alpha
25	239422_at	GPC2	7.40	3.00E−08	Glypican 2 (cerebroglycan)
**ARMS**					
1	211042_x_at	CD146/MCAM	27.68	2.82E−20	Melanoma cell adhesion molecule (MUC18)
2	206633_at	CHRNA1	24.21	7.16E−19	Cholinergic R, nicotinic, alpha 1 (muscle)
3	211237_s_at	FGFR4	19.67	1.00E−16	Fibroblast growth factor receptor 4
4	236517_at	MEGF10	19.19	1.78E−16	Multiple EGF-like-domains 10
5	205327_s_at	ACVR2A	14.78	7.27E−14	Activin A receptor, type IIA
6	205903_s_at	KCNN3	14.00	2.46E−13	K intermediate/small conductance, CNN3
7	206328_at	CDH15	13.85	3.14E−13	Cadherin 15, M-cadherin (myotubule)
8	224182_x_at	SEMA6B	12.15	5.46E−12	Semaphorin 6B
9	239422_at	GPC2	11.89	8.69E−12	Glypican 2 (cerebroglycan)
10	205858_at	CD271/NGFR	11.43	2.03E−11	NGFR, nerve growth factor receptor
11	208299_at	CACNA1I	11.30	2.60E−11	Ca channel, voltage-dep., alpha 1I subunit
12	203510_at	MET	11.07	3.97E−11	Met proto-oncogene
13	221408_x_at	PCDHB12	10.99	4.65E−11	Protocadherin beta 12
14	219692_at	KREMEN2	10.85	6.01E−11	Kringle containing transmembrane protein 2
15	221355_at	CHRNG	10.80	6.64E−11	Cholinergic receptor, nicotinic, gamma
16	212157_at	CD362/SDC2	10.59	1.00E−10	Syndecan 2
17	211693_at	IGHA1	10.43	1.36E−10	Immunoglobulin heavy constant alpha 1
18	212290_at	SLC7A1	10.41	1.43E−10	SLC7 (cationic a.a. transp., y++ system), 1
19	211837_s_at	PTCRA	10.32	1.70E−10	Pre T-cell antigen receptor alpha
20	207555_s_at	TBXA2R	10.29	1.80E−10	Thromboxane A2 receptor
21	230668_at	NKAIN4	10.28	1.83E−10	Na++/K++ transporting ATPase interacting 4
22	222596_s_at	LGR4	10.01	3.11E−10	Leucine-rich repeat-containing GPCR4
23	206128_at	ADRA2C	9.99	3.27E−10	Adrenergic, alpha-2C-, receptor
24	209776_s_at	SLC19A1	9.98	3.31E−10	SLC19 (folate transporter), member 1
25	221450_x_at	PCDHB13	9.96	3.45E−10	Protocadherin beta 13
**ERMS**					
1	211042_x_at	CD146/MCAM	20.87	5.46E−16	Melanoma cell adhesion molecule (MUC18)
2	206633_at	CHRNA1	12.63	1.47E−11	Cholinergic R, nicotinic, alpha 1 (muscle)
3	239422_at	GPC2	11.62	7.31E−11	Glypican 2 (cerebroglycan)
4	208299_at	CACNA1I	11.07	1.83E−10	Ca channel, voltage-dependent, alpha 1I
5	206328_at	CDH15	11.00	2.06E−10	Cadherin 15, M-cadherin (myotubule)
6	224182_x_at	SEMA6B	11.00	2.07E−10	Semaphorin 6B
7	236701_at	GSG1L	10.62	4.00E−10	GSG1-like (has a PMP22 claudin domain)
8	236186_x_at	IL17RE	10.28	7.27E−10	Interleukin 17 receptor E
9	222596_s_at	LGR4	10.06	1.08E−09	Leucine-rich repeat-containing GPCR4
10	214555_at	SSTR5	9.88	1.51E−09	Somatostatin receptor 5
11	207048_at	SLC6A11	9.69	2.14E−09	SLC6 (GABA transporter), member 11
12	236517_at	MEGF10	9.58	2.64E−09	Multiple EGF-like-domains 10
13	205327_s_at	ACVR2A	9.52	2.92E−09	Activin A receptor, type IIA
14	216680_s_at	EPHB4	9.52	2.95E−09	EPH (ephrin) receptor B4
15	237503_at	SLC5A8	9.45	3.32E−09	Solute carrier family 5, member 8
16	209769_s_at	CD42c	9.40	3.71E−09	GP1BB, glycoprotein Ib, beta polypeptide
17	211693_at	IGHA1	9.10	6.57E−09	Immunoglobulin heavy constant alpha 1
18	211837_s_at	PTCRA	9.09	6.67E−09	Pre T-cell antigen receptor alpha
19	209280_at	CD280	9.08	6.75E−09	MRC2, mannose receptor, C type 2
20	209776_s_at	SLC19A1	8.71	1.40E−08	SLC19 (folate transporter), member 1
21	222935_x_at	SLC39A8	8.65	1.59E−08	SLC39 (zinc transporter), member 8
22	207927_at	HTR7	8.59	1.76E−08	5-Hydroxytryptamine (serotonin) receptor 7
23	214546_s_at	P2RY11	8.19	3.96E−08	Purinergic RP2Y, G-protein coupled, 11
24	207555_s_at	TBXA2R	8.12	4.56E−08	Thromboxane A2 receptor
25	234724_x_at	PCDHB18	7.99	6.03E−08	Protocadherin beta 18 pseudogene
**Non_RMS_STS**					
1	244857_at	HBEGF	15.03	5.30E−12	Heparin-binding EGF-like growth factor
2	207555_s_at	TBXA2R	14.55	9.40E−12	Thromboxane A2 receptor
3	208338_at	P2RX3	14.40	1.13E−11	Purinergic R P2X, ligand-gated ion channel, 3
4	210152_at	CD85k/LILRB4	13.51	3.41E−11	Leukocyte Ig-like receptor, subfamily B4
5	211693_at	IGHA1	12.80	8.60E−11	Immunoglobulin heavy constant alpha 1
6	206616_s_a	ADAM22	12.76	9.07E−11	ADAM metallopeptidase domain 22
7	211909_x_at	PTGER3	12.25	1.84E−10	Prostaglandin E receptor 3 (subtype EP3)
8	214546_s_at	P2RY11	12.01	2.57E−10	Purinergic R P2Y, G-protein coupled, 11
9	233171_at	GRIN3A	11.77	3.57E−10	Glutamate receptor, ionotropic, NMDA 3A
10	219516_at	TRPV4	11.57	4.76E−10	Transient R potential cation channel, V4
11	234756_at	CACNG8	11.49	5.41E−10	Ca channel, voltage-dep., gamma subunit 8
21	1554728_at	SLC9A1	11.35	6.55E−10	SLC9 (Na/H exchanger), member 1
13	206729_at	CD30	11.13	9.07E−10	(TNFRSF8, Ki-1)
14	216734_s_at	CD185	11.03	1.07E−09	CXCR5
15	207927_at	HTR7	11.02	1.07E−09	5-Hydroxytryptamine (serotonin) receptor 7
16	208401_s_at	GLP1R	10.99	1.13E−09	Glucagon-like peptide 1 receptor
17	233968_at	CST11	10.96	1.17E−09	Cystatin 11
18	232846_s_at	CDH23	10.88	1.34E−09	Cadherin-like 23
19	233913_at	WFDC10A	10.71	1.73E−09	WAP four-disulfide core domain 10A
20	207048_at	SLC6A11	10.65	1.89E−09	SLC6 (GABA transporter), member 11
21	1567238_at	OR2L2	10.37	2.92E−09	Olfactory R, family 2, subfamily L, member 2
22	221061_at	PKD2L1	10.33	3.10E−09	Polycystic kidney disease 2-like 1
23	244617_at	GPR26	10.18	3.93E−09	G protein-coupled receptor 26
24	216873_s_at	ATP8B2	10.02	5.14E−09	ATPase, Class I, type 8B, member 2
25	211399_at	FGFR2	9.83	6.88E−09	Fibroblast growth factor receptor 2

*Antigens identified in Table 2 were grouped according to functional activity (CD antigens/markers, cell adhesion or cell junction, enzymatic function, growth factor or developmental factor receptor, neurotransmitter receptor, ion channel protein or ion channel regulator, SLC, solute carrier family member, G-protein or G-protein associated, migration or metastasis or cell motility function, tetraspanins, or uncharacterized) in order to give an overview of target types*.

Antigens in the “Cell Adhesion, Cell Junction” group include well-known adhesion family receptor members, like cadherins, cell-to-cell junction associated proteins, and other scavenger or immunoglobulin-related proteins. One would expect that the proteins in this family also would readily have antibodies created to them, and are likely to receive CD designations in the future. This is also likely to be true for the “Growth Factor Receptor/Development Receptor” grouping. These hits also provide ready targets such as the FGFR2, FGFR4, known oncogenes like MET, and the activin and ephrin receptors, that are likely to be linked to the oncogenic activation of the cancer tissues in which they are expressed. The final groups of cell surface proteins that are likely to be targetable are the “Migration/Metastasis/Motility” and the “Enzymatic Function” group. In general these proteins exhibit their activity outside of the cell and mediate interaction of the cell with its surrounding matrix.

Comprising about half the proteins on the list are membrane-expressed proteins to which it cannot be assumed an antibody or CAR exists or that could be readily generated. These groups are the Neurotransmitter Receptors, Ion Channels, Solute Carrier Family, Tetraspanin, and G-protein groups. Although exceptions certainly exist, these membrane proteins interact with small molecules or ions and transport them across the cell membrane, or they co-ordinate other proteins within the membrane. In general they do not evidence a large extracellular component.

**Figure 2:**
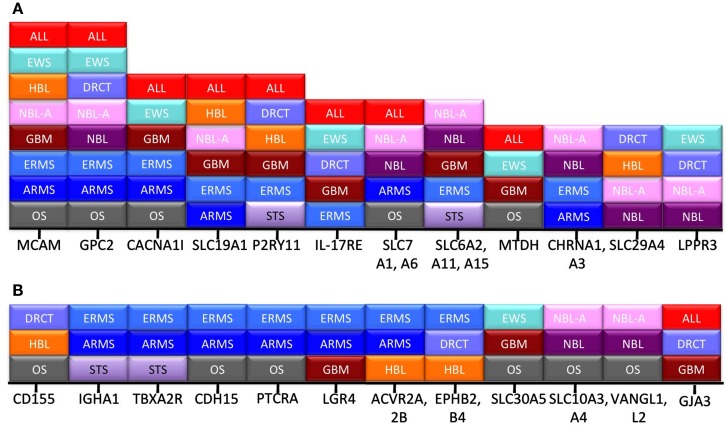
**Frequency of hits across disease category**. Transcripts appearing in the pediatric Top 25 were often shared between disease types. All of the transcripts expressed in more than three **(A)**, or at least three **(B)**, cancer types are listed. The *x*-axis lists the individual gene, and the stacked boxes represent presence of that transcript in the Top 25 for that disease type.

Another way to ascribe relative value to the “hits” we have identified is to determine how broadly they are expressed across the different pediatric cancer types. Figure 2 demonstrates that 12 proteins are expressed in at least three distinct disease categories, and that another 12 transcripts are expressed in more than three disease types. From this view, the highest value targets are MCAM and GPC2, which are expressed in the Top 25 for eight different cancers. Similarly, small molecule transporters like SLC19A1 and neurotransmitter receptors like purogenic G-protein coupled (P2RY11) are prevalent across histologies. Whether the limited size of their extracellular domains will affect the ability to target these antigens with antibody derivatives or CARs is unknown, but their prevalence across disease types makes this an interesting question.

### Target-specific observations

The ability of our algorithm to identify high-value targets is demonstrated by our results for Pre-B ALL, where a number of cell surface molecules currently being targeted in the clinic were identified as hits, Table 2. One of the most important immunotherapeutic trials to date features CAR-modified T-cells specific for CD19, which is the top hit for B_ALL (Kochenderfer et al., 2010; Porter et al., 2011). Another Top 25 hit, CD22, is the target of an antibody-toxin conjugate experimental protocol that continues to show promise for children with ALL (Mussai et al., 2010). Another target, CD79 is also the subject of interest in therapy for B cell malignancy, as antibody-drug conjugates are being developed and tested (Polson et al., 2007). While our bioinformatic approach is validated by these results with ALL, the value of our approach is in the identification of antigens of interest for pediatric solid tumors for which substantial numbers of candidate antigens have not been previously identified.

The two most broadly expressed targets we identified for pediatric solid tumors are melanoma cell adhesion molecule (MCAM, CD146, MUC18), and glypican-2 (GPC2). MCAM and GPC2 are expressed in eight of the 12 tumor types analyzed, Figure 2. The other most commonly expressed adhesion molecule, metadherin (MTDH), is expressed in four of 12 tumor types. MCAM is involved in the pathogenesis of melanoma, breast carcinoma, and other cancers (Wu et al., 2011; Zeng et al., 2012). Both antibody-based and vaccine approaches targeting MCAM/MUC18 have been proposed (Melnikova and Bar-Eli, 2006; Leslie et al., 2007). GPC2 (also know as cerebroglycan) is normally expressed in the developing brain (Stipp et al., 1994). Molecules in the same family, Glypican-3 and Glypican-5, have been described in melanoma, neuroblastoma, and rhabdomyosarcoma (Saikali and Sinnett, 2000; Nakatsura et al., 2004a,b; Williamson et al., 2007). Our report here of the specific expression of Glypican-2 on pediatric tumors should now focus attention on this glypican as well. Metadherin (MTDH) is expressed on hepatocellular carcinoma, may play a role in epithelial-to-mesenchymal transition (Zhu et al., 2011), and plays a role in the metastatic spread of breast cancer to the lung (Brown and Ruoslahti, 2004). These three adhesion receptors are therefore top candidates to explore further as targets in pediatric tumors given their overexpression and the ability to create antibodies against cell surface adhesion receptors.

The largest family of cell surface proteins is the G-proteins (GPCR, approximately 800 members), followed by the solute carrier family (SLC, approximately 380 members; Almen et al., 2009; Hoglund et al., 2011). One of our top hits, SLC19A1 (expressed in ALL, HBL, MYCNA-NBL, GBM, ERMS, and ARMS), is a folate transporter. Unlike the GPI-linked α folate receptor (FOLR1), which is currently the target of antibody-based trials, SLC19A1 (FOLT) is a multi-pass membrane protein that looks very much like an ion channel (Konner et al., 2010). Each of the SLC proteins are regulated in expression, responsive to cellular stress or nutritional requirement, and could serve as suitable immune targets, but little is known about targeting them. The better known GPCR, ion channels, and neurotransmitter receptors we have described have in some cases had antibodies produced against them. In general, producing antibodies, and therefore scFv and CARs, against multi-membrane pass proteins with limited extracellular sequence is more difficult than for proteins with large extracellular domains.

The solid pediatric malignancies we analyzed also preferentially overexpress a number of receptors associated with the activin and ephrin receptor systems. Hits in the activin family include ACVR2A (ERMS, ARMS), ACVR2B (HBL); and in the ephrin family, EPHB2 (DRCT, HBL), EPHB3 (DRCT), EPHB4 (ERMS, DRCT), and EFNB1 (EWS). Activins are growth factors in the TGF-β family that bind dual chain receptors composed of ligand-binding and signaling subunits. The ACVR2 chains have the ability to signal as constitutive kinases and are thought to regulate muscle growth through binding of myostatin (Lee et al., 2005). The ACVR2A/2B expression patterns and their biological activity make them very high-value hits for pediatric oncology. Ephrins function in neuronal development and are divided in to A and B types, as are the receptors that bind to them. The ligand, ephrin-B1, is a transmembrane protein and the receptors we list as Top 25 hits, all in the receptor tyrosine kinase family, are also of the B type. Aberrant expression of these family members has been described in colon carcinoma (Herath et al., 2012). EPHB4 plays a role in vascular development and may participate in tumor metastatic spread (Heroult et al., 2010). Antibodies to EphB4 have been described to inhibit tumor growth (Krasnoperov et al., 2010). Both activin and ephrin receptors are promising for further investigation as cell surface targets in pediatric solid tumors.

Fibroblast growth factor receptors (FGFRs) are of great interest in pediatric oncology, and our bioinformatic hit for FGFR4 in ARMS confirms our previous genomic studies. There are 4 FGFRs that mediate cellular activation by the more than 20 known fibroblast growth factors (Olsen et al., 2003). Alterations or mutation of the FGFRs have been described in a number of cancer types, and they have been proposed as targets for both kinase inhibitor chemotherapy as well as immunotherapy (Wesche et al., 2011). Our group had previously identified FGFR4 as a target in rhabdomyosarcoma using gene expression array analysis and we have demonstrated FGFR4 activity in disease (Taylor et al., 2009). The closely related FGFR2 was also identified as a hit in STS and GBM. FGFR2 has been associated with a number of cancers making both of these FGFRs exciting findings for pediatric oncology (Bai et al., 2010).

### Disease-specific observations

The most intriguing hit for Non-Rhabdomyosarcoma STS is CD30 (TNFRSF8). CD30 is the current target of a number of immunotherapy trials, and our report suggests that these reagents should be evaluated for use in STS (Kasamon and Ambinder, 2008). In DSRCT we find expression of two very exciting hits, poliovirus receptor (PVR)/CD115 and PVR-related 2 (PVRL2)/CD112. Both these adhesion molecules have been described as NK-ligands and should serve as suitable immune targets (Pende et al., 2005). An equally exciting target on DSRCT is PDCD1 (PD1/CD279). The antigen is well-described as an anti-immune effector and efforts to block or target PD1 in cancer are actively underway (Flies et al., 2011).

The immune markers expressed EWS represent a unique group of targets. XG, is part of the Xg blood group antigen, the only other member of which is CD99, a well-known over-expressed protein in EWS, and expression of these antigens is closely linked (Johnson, 2011). Although transcripts are known to be found in non-erythroid tissues not much else is known for this target (Fouchet et al., 2000). Meynet et al. (2010), have also recently described XG as a marker for EWS and demonstrate that its expression is associated with poor outcome. Three cytokine receptors are expressed on EWS: IL17RE, IL27RA, and IL1RAP. A recent mutational study demonstrated that IL27RA has transforming potential, although its expression on non-lymphoid malignancies has not previously been described (Lambert et al., 2011). IL1RAP, Interleukin-1 receptor accessory protein, can exist as a membrane or soluble form and is essential for IL-1 receptor activity. Expression of IL1RAP has been described in endometriosis and various leukemias, notably in leukemia stem cells, making IL1RAP a high-value target for EWS (Jaras et al., 2010; Guay et al., 2011).

Glioblastoma expresses the immune markers in CD73/NT5E (5′ nucleotidase, ecto), and CKLF. CD73 is present on cancer exosomes and has been shown to blunt immune responses (Clayton et al., 2011). CD73 is also a hit for HBL. CKLF, chemokine-like factor 1, binds CCR4 and can be either secreted or membrane-bound and contains a MARVEL transmembrane domain (Chowdhury et al., 2008). MARVELD2 is a transmembrane protein associated with tight junction that serves as a hit for HBL. The wide distribution of these targets requires more developmental work before considering using them as immune targets. GALR2, galanin receptor 2, is a growth factor receptor that also is an interesting GBM target. GALR2 has been proposed as a therapeutic target in head and neck carcinoma and is an active area of study (Kanazawa et al., 2010). GAL2R targeting should be explored in GBM as well.

Osteosarcoma expresses the intriguing hit UL16 binding protein 2 (ULBP2), which is a ligand for the NK cell activation marker NKGD2. To date, ULBP2 has been thought of as a means to alert to immune system due to p53 activation on target cells, and not as a mean of cancer immune escape (Textor et al., 2011). The matrix metalloproteinase MMP14 is known to participate in tumor metastasis and is the subject of intense research activity, making this a high-value target in OS and ASPS (Zarrabi et al., 2011). VASN, vasorin, is a transforming growth factor beta (TGF-β) trap, its biology is regulated by ADAM17, and it appears to play a role in epithelial to mesenchymal transition in other cancer types (Malapeira et al., 2011). Both of these two OS surface targets may play a role in disease progression.

In the analysis of MYCN-Amplified Neuroblastoma (NBL-MYCNA) our algorithm identified one of the most interesting current targets, CD276 (B7-H3). CD276 is the target of a number of immune directed therapies in NBL (Castriconi et al., 2004). The other targets identified for NML-MYCNA and for NB are all of great interest, but CD276 is likely to be a focus of future immune based strategies.

Hepatoblastoma overexpresses ASGR1 asialoglycoprotein receptor 1, and ASGR2. ASGR2 serves as a receptor for a series of glycoproteins including those on the surface of hepatitis virus (Yang et al., 2010). The only report of association with cancer is the reported increase in the rate of cell division of colorectal carcinoma cell lines when grown on ASGR1 coated surfaces, which makes both ASGRs intriguing hits (Fang et al., 2009). Another potential target for HBL is MR1, MHC class I-related, which is also expressed in ASPS. MR1 is an invariant class I MHC molecule known to interact with a subset of T lymphocytes with invariant or restricted TCRs (Gozalbo-Lopez et al., 2009). The expression of MR1 in cancer has not been reported, making this a novel finding. CD301 (CLEC10A, C-type lectin domain family 10, member A) has not been reported in cancer either, but like any adhesion receptor it has the potential to mediate metastasis. Another adhesion receptor hit is CD353, SLAMF8 [signal lymphocyte activation molecule (SLAM), family member 8]. SLAM proteins are in the CD2 family of lymphocyte activation proteins and may also contribute to the activation of cancer cells (Furukawa et al., 2010).

### Alveolar soft part sarcoma

ASPS is a very distinct entity as evidenced by the unique set of antigens we report as top 25 hits, many of which are not shared with other tumors. EPOR, the erythropoietin receptor, was the highest-ranking hit for ASPS. The ETV6-RUNX1 fusion in ALL activates transcription of EPOR and it is likely to contribute a growth signal to leukemia, making this a potential target of interest (Torrano et al., 2011). Two CD antigens that were strong hits for ASPS are also growth factor receptors. Colony-stimulating factor 1/CD115 (CSF1R) modulates a number of myeloid differentiation steps and inhibitors have been designed for a number of disease states (Hume and Macdonald, 2012). CD222/ insulin-like growth factor-2 (IGF2R) has long been recognized as a cancer-expressed protein (Martin-Kleiner and Gall Troselj, 2010). Each of these receptors should be explored as targets in ASPS. Also intriguing is the expression of HLA-G. HLA-G is a class I MHC paralog, normally expressed on placental cells, and its expression has been described on malignant cells, perhaps shielding them from immunosurveillance (Yan, 2011). CD204/macrophage scavenger receptor 1 (MSR1) is another immune molecule expressed on ASPS. Expression of CD204 on tumor stromal macrophages has been associated with aggressiveness in lung cancer (Ohtaki et al., 2010). Thus ASPS provides a number of CD or CD-like antigens that can be targeted by means of immunotherapy.

## Discussion

How a cell surface antigen expressed on a tumor becomes a locus of tumor-protective immune activation, and how this response spares healthy tissue has yet to be fully defined. An antigen may be “revealed” by inhibiting immune checkpoints, or newly recognized as an antigenic target by vaccination (such as with PMSA; May et al., 2011). The key for both approaches is that a response has been induced that now sufficiently differentiates between cancer and normal host tissue. What “sufficient differentiation” is, remains a term in need of clarification. For example, some self-antigens, such as CD20, can readily be targeted, while others, such as HER2, are highly dependent on how they are targeted. The mature B cells compartment is apparently dispensable, as use of anti-CD20 antibody in lymphoma therapy has demonstrated. Once the B cell compartment is no longer targeted by the therapy, the B cell population is then replenished. HER2 (Neu, CD340, ErbB-2) has been effectively targeted in thousands of women with breast cancer using anti-HER2 antibody. However, administration of a T-cell population expressing a HER2-specific CAR resulted in treatment related mortality. In this special instance a large number of anti-HER-2-CAR engineered T-cells presumably bound to the low levels of HER2 present in the lung, and a massive cytokine storm ensued leading to death (Morgan et al., 2010). This event clearly shows that “how” a tumor antigen is targeted by the immune system may be as important as the expression level of that antigen on various tissues. It also illustrates that we have yet to create a universal definition of whether or not it is safe to target a tumor antigen expressed at very low levels on normal tissue. Targeting B cell malignancies has worked so well because the mature B cell compartment that expresses CD20 and CD19 is expendable (Biagi et al., 2007). A database that would begin to define what an “expendable” normal tissue is would be ideal. However, we have yet to even clearly formulate this question with regard to a specific bioinformatic search algorithm. For pediatric cancers, the issue is even more concerning, as certain growth factor receptors may be present on normal tissue that are crucial to development, and would not be part of the “normal” gene expression signature, if it was defined by specimens from adult tissues. Before proceeding with targeting any of the antigens we have identified in this report, we must both confirm expression on tumor and more importantly, confirm as best as we can the lack of expression on “non-expendable” tissues. In this report we identified the major pediatric tumor-associated antigens that are expressed on the plasma membrane, as defined by standard gene chip analysis, statistical analysis, and filtering of annotated attributes. The strength of gene expression profiling is its utility in differential analysis (comparisons of transcript expression in different samples or comparison of tumor versus normal tissue). One potential drawback is that gene chip technology may be skewed, in some instances, toward identification of 3′ transcripts, and thus tumor-specific splice variations or deletions may be missed. Nevertheless studies, including our own, have shown a significant correlation between mRNA and protein levels (Chen et al., 2010). The Affymetrix plus2 array used here attempts to capture multiple independent measurements for each transcript and therefore measures expression of the major transcript as well as other splice variants. However a better more precise but not yet perfect measurement of the expression levels of each transcript is afforded by next generation technologies, e.g., whole RNA sequencing (RNAseq). We are pursuing these measurements, but until complete RNASeq is available for the major pediatric tumor types, along with normal tissue, our approach remains current. Validation with antibody or ligand-based staining for receptors in tumor normal tissues will be needed to definitively credential each candidate transcript.

Ultimately, we plan to continue to refine our definition of which membrane proteins can be safely targeted by CAR or antibody-based therapies, as new analysis techniques emerge. We propose that given the breadth of the antigen list presented here, that a large-scale effort be made to systematically evaluate cell surface targets on pediatric tumors for their potential for immunotherapy. Specifically, antibodies or scFv binding fragments for these antigens need to be screened in a large-scale manner for reactivity to normal tissues. The small size of the overall market in pediatric oncology for these reagents makes it unlikely that industry will engage in this approach on its own. However, given the wealth of genomic and proteomic data being generated, academia can take advantage of these findings and translate them into reagents that can be tested in pre-clinical settings. The analysis framework we present here may also inspire a new look at common adult malignancies, and encourage the development of a new generation of broad-based approaches for identifying immunotherapy targets.

## Conflict of Interest Statement

The authors declare that the research was conducted in the absence of any commercial or financial relationships that could be construed as a potential conflict of interest.

## Footnotes

^1^http://pob.abcc.ncifcrf.gov/cgi-bin/JK

^2^http://home.ccr.cancer.gov/oncology/oncogenomics/

^3^http://www.geneontology.org

^4^http://www.hprd.org/

^5^http://www.cbs.dtu.dk/services/TMHMM/

^6^http://pandeylab.igm.jhmi.edu

^7^http://www.ncbi.nlm.nih.gov/gene
